# Uncertainty Quantification for Space Situational Awareness and Traffic Management †

**DOI:** 10.3390/s19204361

**Published:** 2019-10-09

**Authors:** Samuel Hilton, Federico Cairola, Alessandro Gardi, Roberto Sabatini, Nichakorn Pongsakornsathien, Neta Ezer

**Affiliations:** 1 School of Engineering, Bundoora, RMIT University, Bundoora, VIC 3083, Australia; sam.hilton@rmit.edu.au (S.H.); alessandro.gardi@rmit.edu.au (A.G.); s3679479@student.rmit.edu.au (N.P.); 2 Politecnico di Torino – DIMEAS, 10129 Turin, Italy; s243849@studenti.polito.it; 3 Northrop Grumman Corporation, 1550 W. Nursery Rd, Linthicum Heights, MD 21090, USA; neta.ezer@ngc.com

**Keywords:** Space Traffic Management, Cyber-Physical Systems, Resident Space Object, Space-Based Surveillance, Radar Performance, Gauss–Helmert Method, Space Situational Awareness, Uncertainty Quantification, Covariance Realism, Cognitive Human-Machine Interaction

## Abstract

This paper presents a sensor-orientated approach to on-orbit position uncertainty generation and quantification for both ground-based and space-based surveillance applications. A mathematical framework based on the least squares formulation is developed to exploit real-time navigation measurements and tracking observables to provide a sound methodology that supports separation assurance and collision avoidance among Resident Space Objects (RSO). In line with the envisioned Space Situational Awareness (SSA) evolutions, the method aims to represent the navigation and tracking errors in the form of an uncertainty volume that accurately depicts the size, shape, and orientation. Simulation case studies are then conducted to verify under which sensors performance the method meets Gaussian assumptions, with a greater view to the implications that uncertainty has on the cyber-physical architecture evolutions and Cognitive Human-Machine Systems required for Space Situational Awareness and the development of a comprehensive Space Traffic Management framework.

## 1. Introduction

The ever-increasing number of Resident Space Objects (RSO) is strongly highlighting the need for an evolution from traditional Space Situational Awareness (SSA) capabilities towards Space Domain Awareness (SDA) [1,2]. Analogous to its atmospheric counterpart (i.e., Air Domain Awareness) SDA aims to elevate current SSA capabilities through the dissemination of confidence-building measures, necessary to reliably estimate the future states of RSO’s with the aim of optimal coordination and accommodation in a future Space Traffic Management (STM) system. Confidence-building measures ideally encapsulate the functional characteristics of an RSO (e.g., shape and size), and, when possible, mission objectives and planned operational activities of active spacecraft. Even so, an increase alone in the available data will not solve the current issues of RSO ambiguity and collision avoidance subjectivity – contextualizing information must be used together with transparent and traceable Time and Space Position Information (TSPI) reflective of sensor performance. Cooperative RSO equipped with TSPI enabling systems such as GNSS and data sharing capabilities equivalent to Automatic Dependent Surveillance Broadcast (ASD-B) system will be an important aspect in managing uncertainty in the on-orbit environment. Nonetheless, due to the inherently high threat of space debris, it is imperative that the notion of transparent and traceable TSPI information extends to classification of non-cooperative RSOs. In most cases, the position of large orbiting object (>10 cm) can be predicted with reasonable uncertainty, based upon data accrued by the SSA Space Surveillance and Tracking (SST) segment and other non-government owned ground-based sensors (Table 1).

Although ground-based systems will continue to be a vital aspect in filling the SST role, the feasibility of Space-Based Space Surveillance (SBSS) is being explored to monitor the Low Earth Orbit (LEO) [3] and Geosynchronous Orbit (GEO) regions [4,5].due to the advantage of persistent coverage of smaller sized RSOs (<10 cm) elusive to traditional ground-based systems. Understandably spaceborne tracking of RSOs is not a trivial matter [6,7] – effective coverage of the space environment will require constellations of SBS platforms each under complex tasking regimes to overcome performance and physical constraints [8]. Given that true collisions are a rare space event, the cost of platforms devoted to debris detection may exceed their benefit given that there would be minimal power available for mission-oriented payloads. This is especially the case for spaceborne radar due to the higher power requirements associated with this sensor. For future SBSS platforms to be commercially and economically viable, it is imperative that a low size weight, power, and cost (SWaP-C) approach must be taken. Regardless of the sensor suite chosen, SBSS platforms will be subject to dynamic positional errors from onboard TSPI/Navigation systems, in contrast to traditional ground-based systems that perform observations from accurately surveyed locations. As such, there is a strong case for analysis on the effect of initial RSO position estimation based on both navigation and tracking system errors. Nevertheless, in both ground-based and SBSS applications, a sensor-focused approach must be taken to establish an unambiguous initial estimate of RSO uncertainty.

In the context of SSA, realistic orbital uncertainty directly underpins the effectiveness of operational activities that include the following: RSO Orbit determination, data or track association/correlation, maneuver detection, the computation of the probability of collision for conjunction assessment and sensor management [9,10]. Within the SSA/STM research community, Sensor Management is comprised of Sensor Tasking (ST) and Sensor Scheduling (S2). ST is defined as the generation of a set of tasks that a sensor or sensor network is intended to accomplish. On the other hand, Sensor Scheduling (S2) refers to the specific decisions involved in the tasks, to include the exact definition of when and where a sensor is to be used. ST and S2 are typically the constituting elements of an optimal control problem to dynamically assign the available sensor resources (ST) to accomplish specific SSA tasks (S2) [10]. Most importantly, these processes require the knowledge of how the uncertainty of RSOs will propagate over time subject to orbital dynamics and perturbations from residual atmospheric drag, solar radiation pressure, non-spherical Earth, and other celestial bodies. Largely, Orbital uncertainty propagation methodologies can be grouped under either Linear and Non-Linear Methods [11]. Typical Non-Linear methods include Unscented Transformations, Polynomial Chaos Expansions, and Fokker Plank Methods. Undoubtedly, these methods can capture well the non-linear growth of RSO uncertainty subject to orbital dynamics, however, can be computationally burdening due to the high dimensionality of the problem especially for longer propagation periods. The mathematical derivation of these methods is beyond the scope of this paper, the reader is referred to reference [11]. A popular alternative is to construct a linearized model of the dynamics so that the uncertainty about an RSO can be propagated in a computational efficient manner. However, linearized propagation methods are not without shortcomings -which if not explicitly quantified have significant implications on the realism of subsequent analysis made based on the estimated uncertainty. Uncertainty quantification is defined as follows: *“The process of determining the various sources of errors and uncertainties, properly characterizing these errors and uncertainties, and the roll up of these in the prediction of the quantities of interest”* [10]. In the context of linearized uncertainty propagation methods, two fundamental assumptions are made:

1. A linearized model sufficiently approximates the dynamics of neighboring trajectories with respect to a nominal trajectory.
2. The uncertainty can be completely characterized by a Gaussian probability distribution.

To quantify the uncertainty, we are interested in testing the uncertainty realism, which under Gaussian assumptions (linear), coincides with covariance realism – the characterization (size, shape, and orientation) of the (gaussian) uncertainty of the RSO in question. Various covariance realism tests and metrics have been used to assess realism with a primary focus on determining the length of Gaussian validity, i.e., the amount of time (commonly described in orbital periods) a linearized uncertainty propagation method can be used to confidently and accurately describe the estimated position uncertainty of an RSO. Nonetheless, by reiteration of the above assumption, linear propagation methods are only valid if the initial RSO uncertainty (input) is in fact Gaussian. This presents the necessary case of applying covariance realism testing to the sensor level with the aim of quantifying tracking and navigation system performance characteristics that support, or not, a linearized (Gaussian) method to describe initial RSO position uncertainty. By taking a sensor level perspective to model tracking and navigation system uncertainty, this paper addresses two SST representative case studies.-traditional ground-based radar and proposed Millimetre Wave (MMW) Space-Borne Radar (SBR) for the tracking of space debris aboard larger spacecraft platforms such as the International Space Station (ISS) and future space transportation vehicles [6,12].

### Fundamental Role of Uncertainty in SSA and STM Human-Machine System Integration

**Figure 1:**
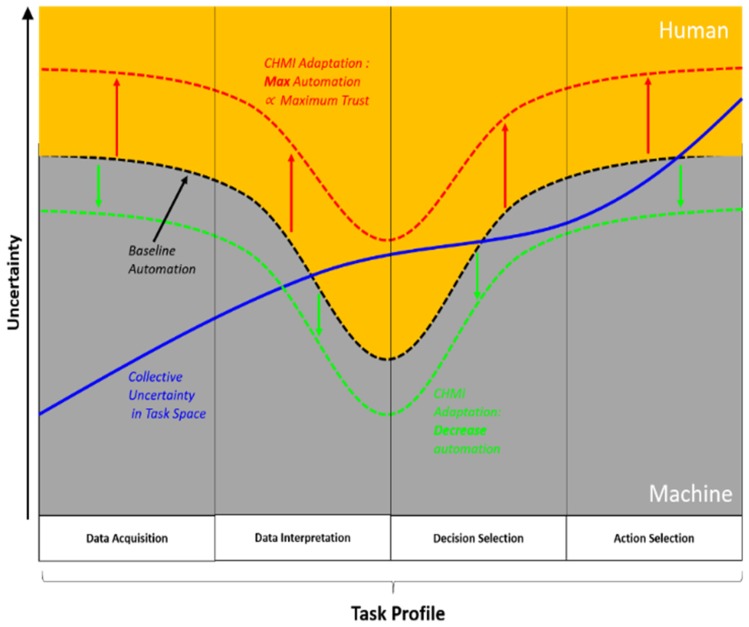
Conceptual Adaptive Human Machine Decision Space.

The accurate and timely quantify uncertainty about cooperative and non-cooperative RSOs will be a key driver in determining a baseline for cyber-physical system autonomy within an SSA and greater STM framework. In other words, the *trustworthiness* of the machine to perform tasks under an explicit level of collective uncertainty can be understood. Equally, the SSA and STM decision space should not bias automation over the human, where explicit considerations regarding continued situational awareness, attention, vigilance as well as expected cognitive ability of the operator must be made. To realize the concept of a fully integrated Human-Machine cyber physical system, and in turn achieve an optimal teaming between STM ground station operators and system autonomy, future decision support systems must build upon traditional representations of uncertainty towards the use of extended methods [13,14] that meet well-defined mission goals as well as adopting an architecture that supports Cognitive Human-Machine Interfaces and Interactions (CHMI^2^) [15]. The concept of CHMI^2^ capitalises and builds upon the considerable advances in aerospace avionics human factors [16,17], which was presented in detail in [18,19]. Nonetheless, the importance of human machine integration is now gaining well-deserved attention in the Space sector [20,21,22,23,24] due to the mission and safety critical tasks performed by space analysts. Figure 1 illustrates the concept of variable automation space (dashed black, red and green lines) throughout four stages of human cognitive processing based on the Parasuraman et al. task-based profile: Data Acquisition, Data Interpretation, Decision Selection and Action Selection [25]. The automation level line lies at the boundary between human (dark yellow) and machine (grey) involvement. Another important aspect represented is the magnitude of uncertainty (blue solid line), which in this example is depicted as monotonically growing across the four stages. By providing the feedback loop between the operator and the system through a suite of physiological sensors, the CHMI^2^ concept enables the automation curve to be dynamically adjusted based on the operator cognitive state. The automation space is dictated by the degree of collective uncertainty in the environment and can be classified under two categories: Aleatory and Epistemic uncertainty (Table 2) [26].The focus of this article is on addressing *aleatory* uncertainty within the orbital environment, namely parameter value(measurement uncertainty) and model-based uncertainty (covariance realism).

## 2. Methods and Models

Firstly, radar tracking (TRK) and navigation (NAV) error models are developed, including common transformations required to represent the uncertainty in a convenient frame. Attention is then turned to methods to assess the “realism” of the uncertainty under Gaussian assumptions using two common approaches - the Average Mahalanobis Distance (AMD) statistic metric and Cramer–von Mises (CVM). A Monte-Carlo framework is then presented to obtain the required Empirical Distribution of both tracking and navigation uncertainty.

### 2.1. Tracking and Navigation System Error Models

The SNR (Signal to Noise Ratio) of the radar is a key measure of its performance, which is defined as the ratio of signal power to noise power at the output of the radar receiver.

(1) $$SNR=\frac{P_{r}}{P_{n}}=\frac{P_{p}G_{t}\sigma A_{r}\tau }{{\left(4\pi \right)}^{2}R^{4}kFT_{0}L}$$

where:

$P_{p}$: Peak transmitted power [W]
$G_{t}$: Radar transmit antenna gain (power ratio)
$A_{r}$: Radar receive antenna effective aperture area [$m^{2}$]
*σ*: Target radar cross-section (RCS) [$m^{2}$]
*R*: Range from radar to target [m]
*F*: Noise figure of the receiver subsystem
*L*: Radar system losses
*T*_0_: Standard Temperature [290 K]
*k*: Boltzmann’s constant [1.38064852 × $10^{-23}$ $m^{2}$Kg $s^{-2}K^{-1}$]
τ: Radar pulse duration [sec]

**Figure 2:**
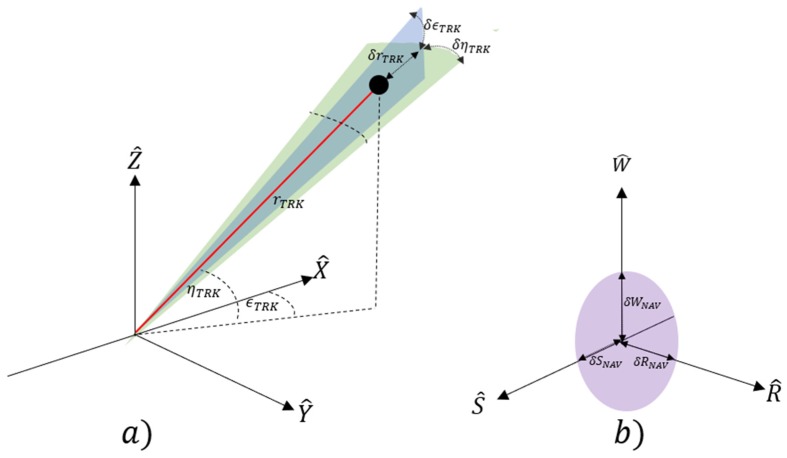
Generic Tracking (**a**) and Navigation (**b**) RSW coordinate systems detailing corresponding error geometry.

Target state vector information is measured relative to the radar site in a spherical coordinate system in range, elevation and azimuth ($r_{RDR},\;\eta_{RDR},\;\epsilon_{RDR}$ respectively) (Figure 2a) The measurements in each of the elements are prone to specific error sources that include the following [27]:

(2) $$\sigma_{r_{RDR}}{}^{2}=\sigma_{RN}^{2}+\sigma_{RF}^{2}+\sigma_{RB}^{2}$$

where $\sigma_{RN}$ is an SNR dependent random range measurement error, which can be calculated as:

(3) $$\sigma_{RN}=\frac{c}{2B\sqrt{2\left(SNR\right)}}$$

where, B is waveform bandwidth, c is the speed of light and signal to noise ratio (SNR). $\sigma_{RF}$ is a random measurement error having fixed standard deviation, due to noise sources in the latter stages of the radar receiver. $\sigma_{RB}$ is a range bias error associated with the radar calibration and measurement process. We assume the Zero-mean condition, so. $\sigma_{RB}$ and $\sigma_{RF}$ are equal to zero.

Radar angular measurements are commonly made using monopulse receive antennas that provide a difference pattern characterized by a deep null on boresight. The difference pattern formed by these beams may be used to measure target angular position with a single signal transmission. The measurement accuracy in each angular coordinate is characterized by the RMS of the SNR dependent random angular measurement error, angular bias, and random measurement error. As with the range error, we assume angular bias and random measurement error to be 0 under the Zero-mean condition

(4) $$\sigma_{\epsilon_{RDR}}{}^{2}=\sigma_{AN_{\epsilon }}^{2}+\sigma_{AF_{\epsilon }}^{2}+\sigma_{AB_{\epsilon }}^{2}$$

(5) $$\sigma_{\eta_{RDR}}{}^{2}=\sigma_{AN_{\eta }}^{2}+\sigma_{AF_{\eta }}^{2}+\sigma_{AB_{\eta }}^{2}$$

As with the range errors, the SNR dependent error dominates the radar angle error:

(6) $$\sigma_{AN}=\frac{\vartheta }{k_{m}\sqrt{2\left(SNR\right)}}$$

where: ϑ is the radar beamwidth in the angular coordinates and $k_{m}$ is the monopulse pattern difference slope.

The RSW satellite coordinate system is chosen to express position uncertainty of RSO. At the time of observation, we assume that the nominal spacecraft position (SP) is centered at the origin of the RSW axis The Radial (R) axis always points from the earth centre along the radius vector towards the satellite. The S-Axis is pointed tangentially to the track’s direction, where, in the case of elliptical orbits, it is only parallel to the velocity vector at apogee and perigee. The W (cross-track) axis is normal to the orbital plane and completes the right-hand triad (Figure 2b). The coordinate system can then be constructed through the following unit vector approach [28]:

(7) $$\hat{R}=\frac{R_{ECI}}{\left|R_{ECI}\right|}$$

(8) $$\hat{W}=\frac{R_{ECI}\times V_{ECI}}{\left|R_{ECI}\times V_{ECI}\right|}$$

(9) $$\hat{S}=\hat{W}\times \hat{R}$$

The transfer matrix(s) between the RSW and ECI coordinate systems is then following:

(10) $$M_{RSW\to ECI}=\left[\hat{R\;}\hat{S\;}\hat{W}\right]$$

(11) $$M_{ECI\to RSW}={\left[\hat{R\;}\hat{S\;}\hat{W}\right]}^{T}$$

Positional errors from the on-board navigation system are then expressed as deviations, $\delta X_{NAV}$, from the origin of the axis, defined as the difference between the true state, $X_{NAV}$, and the nominal state ${\bar{X}}_{NAV}$ under the zero mean

(12) $$\delta X_{NAV}=X_{NAV}-{\bar{X}}_{NAV}$$

(13) $$X_{NAV}=\left[\begin{array}{c} R_{T}\\ S_{T}\\ W_{T} \end{array}\right],{\bar{X}}_{NAV}=\left[\begin{array}{c} R_{N}\\ S_{N}\\ W_{N} \end{array}\right]$$

Navigation uncertainty is assumed to be Gaussian, and can then be expressed in terms of covariance, where the assumption of zero main is made:

(14) $$Q_{NAV}{}^{RSW}=E\left[\delta X_{NAV}\delta X_{NAV}{}^{T}\right]={\left[\begin{array}{ccc} \sigma_{R_{NAV}}{}^{2} & 0 & 0\\ 0 & \sigma_{S_{NAV}}{}^{2} & 0\\ 0 & 0 & \sigma_{W_{NAV}}{}^{2} \end{array}\right]}^{}$$

Similarly, tracking measurement errors are expressed as measurement deviations in the spherical dimension, $\delta X_{TRK},$ defined as the difference between the true state, $X_{TRK}$, and the nominal state ${\bar{X}}_{TRK}$ of the RSO [29].

(15) $$\delta X_{TRK}=X_{TRK}-{\bar{X}}_{TRK}$$

(16) $$X_{TRK}=\left[\begin{array}{c} r_{T}\\ \epsilon_{T}\\ \eta_{T} \end{array}\right],{\bar{X}}_{TRK}=\left[\begin{array}{c} r_{N}\\ \epsilon_{N}\\ \eta_{N} \end{array}\right]$$

The tracking error of the radar is then expressed in terms of covariance:

(17) $$Q_{TRK}{}_{SPH}^{RDR}=E\left[\delta X_{TRK}\delta X_{TRK}{}^{T}\right]={\left[\begin{array}{ccc} \sigma_{r_{TRK}}{}^{2} & 0 & 0\\ 0 & \sigma_{\epsilon_{TRK}}{}^{2} & 0\\ 0 & 0 & \sigma_{\eta_{TRK}}{}^{2} \end{array}\right]}^{}$$

### 2.2. Transformation of Uncertainty to Common Coordinate System

To combine NAV & TRK uncertainty from the spacecraft and tracked RSO both covariance matrices must belong to the same coordinate frame. In this case, a transformation from the spherical to the Cartesian system must be performed. Position and velocity measurements (and uncertainty) should be expressed in a reference frame that is most convenient to the user, where in this case a Cartesian Earth-Centered Inertial (ECI) frame is chosen. As such, the navigation covariance matrix must be transformed to the Cartesian Earth-Centred inertial (ECI) frame. As this is a linear process (cartesian to cartesian), a simple coordinate transformation can be applied to the covariance matrix. Following the derivation of the RSW to ECI transformation matrix previously described we can write:

(18) $$Q_{NAV}{}_{CART}^{ECI}=M_{RSW\to ECI}\;Q_{NAV}{}_{CART}^{RSW}\cdot M_{RSW\to ECI}{}^{T}$$

(19) $$Q_{NAV}{}_{CART}^{ECI}=\left[\begin{array}{ccc} \sigma_{x_{NAV}}{}^{2} & \sigma_{xy_{NAV}} & \sigma_{xz_{NAV}}\\ & \sigma_{y_{NAV}}{}^{2} & \sigma_{yz_{NAV}}\\ sym & & \sigma_{z_{NAV}}{}^{2} \end{array}\right]$$

In contrast, the tracking covariance matrix is expressed in a spherical coordinate system within the radar frame. This requires both a transformation from spherical to Cartesian system and then a translation to the ECI frame. As the transformation between these systems is nonlinear, a basic coordinate transformation is not sufficient, and mathematical tools such the Jacobian of the spherical to Cartesian transformation matrix must be calculated to linearize the process. The spherical to Cartesian Jacobian (D) is expressed as the following, where c, s and represent the cosine and sine of the radar angular measurements.

(20) $$D=\left[\begin{array}{ccc} -c\;\epsilon_{TRK}\;c\;\eta_{TRK} & r_{TRK}\;c\;\epsilon_{TRK}\;s\;\eta_{TRK} & r_{TRK}\;s\;\epsilon_{TRK}\;c\;\eta_{TRK}\\ \;c\;\epsilon_{TRK}\;s\;\eta_{TRK} & r_{TRK}\;c\;\epsilon_{TRK}\;c\;\eta_{TRK} & -r_{TRK}\;s\;\epsilon_{TRK}\;s\;\eta_{TRK}\\ \;s\;\epsilon_{TRK} & 0 & r_{TRK}\;c\;\epsilon_{TRK} \end{array}\right]$$

The transformation from spherical tracking error matrix in Radar coordinate system to the Cartesian ECI is then given by the following:

(21) $$Q_{TRK}{}_{CART}^{ECI}=\left(M_{RDR\to ECI}\cdot D\right)\cdot Q_{TRK}{}_{SPH}^{RDR}\cdot {\left(M_{RDR\to ECI}\cdot D\right)}^{T}$$

(22) $$Q_{TRK}{}_{CART}^{ECI}=\left[\begin{array}{ccc} \sigma_{x_{TRK}}{}^{2} & \sigma_{xy_{TRK}} & \sigma_{xz_{TRK}}\\ & \sigma_{y_{TRK}}{}^{2} & \sigma_{yz_{TRK}}\\ sym & & \sigma_{z_{TRK}}{}^{2} \end{array}\right]$$

where $M_{RDR\to ECI}$ is the transformation matrix from the chosen Radar (TRK) coordinate frame to the ECI frame. The covariance matrix of both the navigation and tracking can now be expressed geometrically as an ellipsoid centered about the nominal position in the ECI Frame. Due to the transformation and translations between the Radar Spherical and Cartesian coordinate systems to the ECI frames, the covariance terms within the error matrix (off-diagonal) are now non-zero. The geometric interpretation of $Q_{TRK}{}_{}^{ECI}$ now requires that the ellipsoid considers both the variances about the principal axis but also the rotation within the cardinal system (ECI).

### 2.3. Assessing Covariance Realism at the Sensor Level

Within the SSA/Astrodynamics community, the assessment of covariance realism (also known as covariance consistency) has been predominately focused upon identifying the point at which Gaussian assumptions in the propagation of orbital uncertainty breakdown. As discussed, this paper interested at applying this approach to the sensor level and in turn validating when gaussian assumptions of navigation and tracking error break down. In doing so, 2 commonly used statistical metrics and goodness of fit tests have been adopted. The Mahalanobis distance [30] provides a convenient metric for testing covariance realism, where a set of empirically generated points, $x_{\mathrm{mc}}$, from the measurement model are tested to see if it corresponds to the gaussian distribution defined by a covariance matrix P centered about the truth state, $x_{\mathrm{truth}}$, of the target. The squared Mahalanobis distance between the estimated orbit state and the truth target is defined as:

(23) $$ℳ(\left(x_{\mathrm{mc}},x_{\mathrm{truth}},P\right)={\left(x_{\mathrm{mc}}-x_{\mathrm{truth}}\right)}^{T}P^{-1}\left(x_{\mathrm{mc}}-x_{\mathrm{truth}}\right)$$

The expected value of ℳ is *n*, where *n* is the dimension of the state vector $x_{\mathrm{truth}}$, which in the case of a cartesian coordinate system corresponds to 3. As an uncertainty realism metric, one can consider the values of $\bar{ℳ}$, averaged over at each observation condition. Let ${ℳ}^{\left(i\right)}$ be the uncertainty realism metric computed in the *i*-th Monte Carlo trial. Let *k* be the total number of independent trials.

(24) $$\bar{ℳ}=\frac{1}{nk}\overset{k}{\underset{i=1}{\sum }}{ℳ}^{\left(i\right)}$$

A stronger test for uncertainty realism is to consider the statistical distribution determined from the measurement model in the form of a physics-based Monte-Carlo simulation. As such, the second covariance realism metric test used is the Cramer–von Mises goodness of fit test statistic [9,10]. This test permits to verify the consistency of the sample and test how well the theoretical Gaussian distribution fits the empirical distribution. The Cramer–von Mises (CVM) test is based in a statistic of the type

(25) $$Q_{k}=\int_{-\infty }^{+\infty \;\;\;}{\left[F_{n}\left(x\right)-F^{*}\left(x\right)\right]}^{2}\varphi \left(F\left(x\right)\right)dF^{*}\left(x\right)$$

where $F^{*}\left(x\right)$ is the cumulative distribution function (CDF) of the Mahalanobis distance ℳ and $F_{n}\left(x\right)$ is the Empirical CDF of the AMD representing the *n* degree of freedom system being analyzed. Where the results are from a Monte Carlo simulation of the measurement error model with N samples. Specializing to φ(F(x))=1, the CVM test is then calculated by:

(26) $$Q_{k}=\frac{1}{12N}\overset{N}{\underset{i=1}{\sum }}{\left[\frac{2i-1}{2N}-F\left({ℳ}^{\left(i\right)}\right)\right]}^{2}$$

Sorting the Mahalanobis squared distance of the samples, ${ℳ}^{\left(i\right)}$, from the smallest to largest, $F\left({ℳ}^{\left(i\right)}\right)$ can be obtained by:

(27) $$F\left({ℳ}^{\left(i\right)}\right)=erf\left(\sqrt{\frac{{ℳ}^{\left(i\right)}}{2}}\right)-\sqrt{\frac{2\;{ℳ}^{\left(i\right)}}{\pi }}e^{-\frac{{ℳ}^{\left(i\right)}}{2}}$$

Given a significance level α, one can derive a two-sided 100(1 − α)% confidence interval for the distribution $\bar{ℳ}\left(n\right)$. As with the averaged Mahalanobis distance (AMD), the acceptable degree of the CVM metric is determined by defining a confidence level. Table 3 outlines the acceptable ranges of the CVM and AMD for a commonly selected confidence level for measurement models of dimension 3.

As described the Squared Mahalanobis Distance Metric and the Cramer–von Mises distribution matching test require the generation of an Empirical Distribution. In doing so, a measurement model using the calculated uncertainty of the radar and tracking error models is constructed, generating N observation samples about the nominal measurement. Under the assumption that each measurement variable is independent (non-correlated):

The navigation Error contribution, (considered only for SBSS platform), is given by:

(28) $$\left\{\begin{array}{c} R_{N}=\sigma_{R_{NAV}}N\\ S_{N}=\sigma_{S_{NAV}}N\\ W_{N}=\sigma_{W_{NAV}}N \end{array}\right\}$$

The tracking contribution is given by:

(29) $$\left\{\begin{array}{c} r_{T}=r_{0}+\sigma_{r_{TRK}}N\\ \epsilon_{T}=\epsilon_{0}+\sigma_{\epsilon_{TRK}}\;N\\ \eta_{T}=\eta_{0}+\sigma_{\eta_{TRK}}N \end{array}\right\}$$

The total uncertainty about the object when tracked from the space-based platform is then described by:

(30) $$\left\{\begin{array}{c} R_{T}=R_{N}+r_{T}cos\left(\eta_{T}\right)\;cos\left(\epsilon_{T}\right)\\ S_{T}=S_{N}+r_{T}cos\left(\eta_{T}\right)\;sin\left(\epsilon_{T}\right)\\ W_{T}=W_{H}+r_{T}sin\left(\epsilon_{T}\right) \end{array}\right\}$$

Under the assumption the position of observation is well known and therefore the error is negligible, the total uncertainty from the ground station is:

(31) $$\left\{\begin{array}{c} S_{T}=r_{T}cos\left(\eta_{T}\right)cos\left(\epsilon_{T}\right)\\ E_{T}=r_{T}cos\left(\eta_{T}\right)sin\left(\epsilon_{T}\right)\\ Z_{T}=r_{T}sin\left(\epsilon_{T}\right) \end{array}\right\}$$

## 3. Ground-Based Tracking Scenario

**Figure 3:**
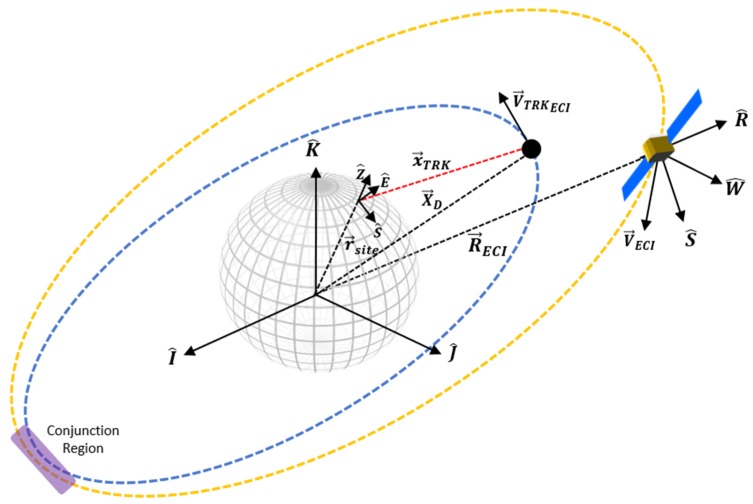
Illustration of ground-based tracking scenario and subsequent conjunction region with operational satellite.

The aim of the first case studies is to apply the above framework for the typical scenario of RSO tracking from a radar ground station for the practical purpose of identification and assessment of a potential collision with an operational spacecraft (Figure 3). Typically ground-based tracking stations utilize the South East Zenith Topocentric Horizon Coordinate frame (SEZ). The SEZ coordinate system is defined for a given longitude and latitude at a local sidereal time and rotates with the site where the local horizon forms the fundamental plane. The S axis points due South from the site, The E axis points East from the site and the Z axis (Zenith) points radially outward from the site along the site position vector from the ECI origin.

The coordinate system is constructed using the site position vector, ${\vec{r}}_{SITE}$, in ECI frame:

(32) $$\hat{Z}=\frac{{\vec{r}}_{SITE}}{\left|{\vec{r}}_{SITE}\right|}$$

(33) $$\hat{E}=\hat{K}\times \hat{Z}$$

(34) $$\hat{S}=\hat{E}\times \hat{Z}$$

The transfer matrix between the RSW and ECI coordinate systems is then following:

(35) $$M_{SEZ\to ECI}=\left[\hat{S\;}\hat{E}\;\hat{Z}\right]$$

Within the SEZ coordinate frame the tracked RSO range, azimuth and elevation (ρ,ϵ,η) and their derivates are measured where then by implementing the SITE-TRACK algorithm [28] the position ${\vec{X}}_{TRK}$ and the velocity ${\vec{V}}_{TRK}$ in the ECI frame can be determined.

(36) $${\vec{X}}_{TRK}={\vec{r}}_{SITE}+M_{SEZ\to ECI}\left[\begin{array}{c} \rho cos\left(\eta \right)cos\left(\epsilon \right)\\ \rho \;cos\left(\eta \right)sin\left(\epsilon \right)\\ \rho \;sin\left(\eta \right) \end{array}\right]$$

(37) $${\vec{V}}_{TRK}=M_{SEZ\to ECI}\;{\vec{v}}_{SEZ}+\vec{\omega }\;x\;{\vec{X}}_{TRK}$$

where: ${\vec{v}}_{SEZ}$ is the velocity vector determined from observations at the site in SEZ coordinate frame and $\vec{\omega }$ is the Earth’s rotation vector. Based on the calculated radar performance parameters using radar error equations (Equations (2), (4) and (5)), it is then necessary to transform the TRK uncertainty ($\sigma_{r_{TRK}}$, $\sigma_{\epsilon_{TRK}}$, $\sigma_{\eta_{TRK}}$) into the ECI coordinate system, $Q_{TRK}{}_{CART}^{ECI}$ as described in Section 2.3. Assuming the velocity measurement error to be zero we obtain the following 3 × 3 Covariance matrix for each observation.

(38) $$Q_{TRK}{}_{CART}^{ECI}=\left(M_{SEZ\to ECI}\cdot D\right)\cdot Q_{TRK}{}_{SPH}^{SEZ}\cdot {\left(M_{SEZ\to ECI}\cdot D\right)}^{T}$$

(39) $$Q_{TRK}{}_{CART}^{ECI}=\left[\begin{array}{ccc} \sigma_{x_{TRK}}{}^{2} & \sigma_{xy_{TRK}} & \sigma_{xz_{TRK}}\\ & \sigma_{y_{TRK}}{}^{2} & \sigma_{yz_{TRK}}\\ sym & & \sigma_{z_{TRK}}{}^{2} \end{array}\right]$$

Subject to a linearized propagation method, the dynamic evolution of the RSO uncertainty $Q_{TRK}{}_{CART}^{ECI}$ now be estimated. As this paper is focused on the sensor level analysis, the full derivation of this technique beyond scope and as such the reader is referred to [11] and [31] for this additional framework. As previously discussed, the propagation of uncertainty is a fundamental aspect of SSA, as it allows the determination of probability of collision between two RSO’s if a close approach is predicted from each nominal RSO trajectory, a region known as the “Conjunction Region” is then defined. The covariance matrix’s that describe the propagated position uncertainty of the tracked RSO (${Q^{\prime }}_{TRK}$) and the spacecraft (${Q^{\prime }}_{NAV}$) are then summed together and represented as an ellipsoid typically centered on the nominal position of the tracked (non-cooperative) RSO. Measurements are uncorrelated, variance and covariance terms can be summed directly [32].

(40) $$Q_{TOT}={Q^{\prime }}_{TRK}+{Q^{\prime }}_{NAV}$$

(41) $$Q_{TOT}={\left[\begin{array}{ccc} \sigma_{x_{}}{}^{2} & \sigma_{xy_{}} & \sigma_{xz_{}}\\ & \sigma_{y_{}}{}^{2} & \sigma_{yz_{}}\\ sym & & \sigma_{z_{}}{}^{2} \end{array}\right]}^{ECI}$$

**Figure 4:**
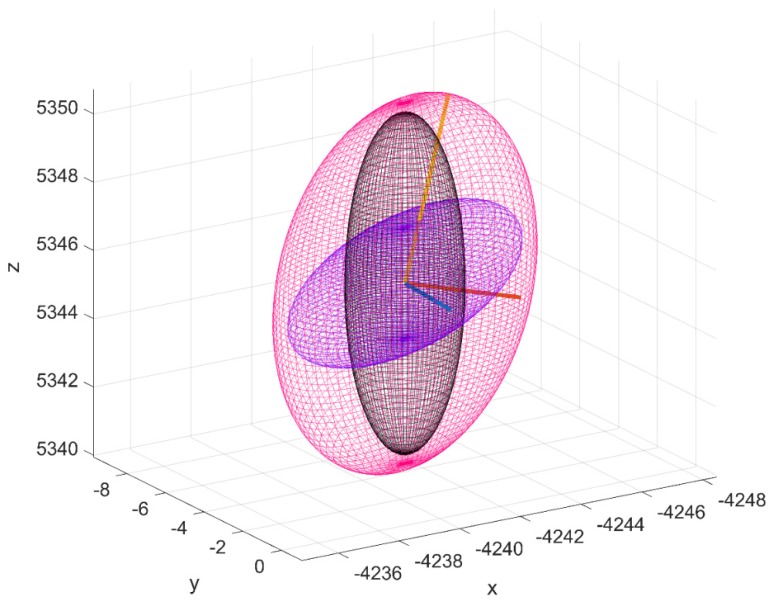
Conceptual illustration of navigation (**purple**) tracking (**black**), total uncertainty volume (**magenta**) and major (**orange**), semi-major (**red**) and minor axis (**blue**) of total uncertainty volume.

Following from reference REF $Q_{TOT}$ now provides a convenient form to analyze the probability of collision between the tracked and operation RSO. Figure 4 illustrates the concept of individual navigation and tracking uncertainty’s volume and the resultant combined ellipsoid.

## 4. Space-Based Surveillance Scenario

**Figure 5:**
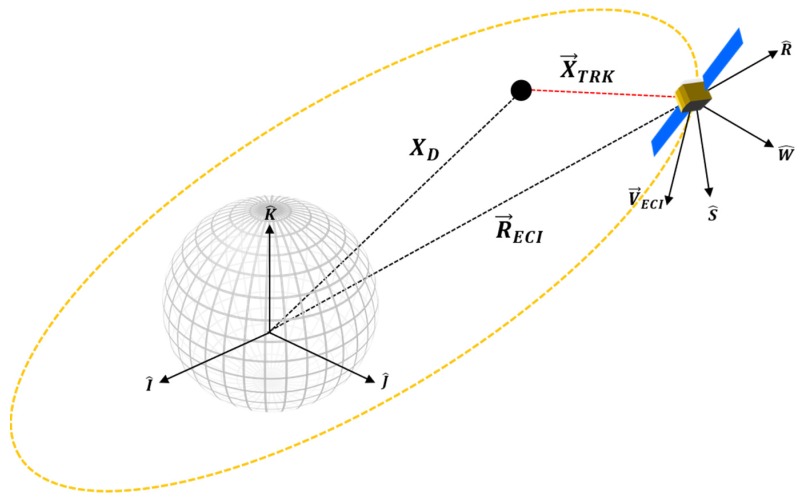
Reference geometry of the non-cooperative tracking and RSW coordinate system.

**Figure 6:**
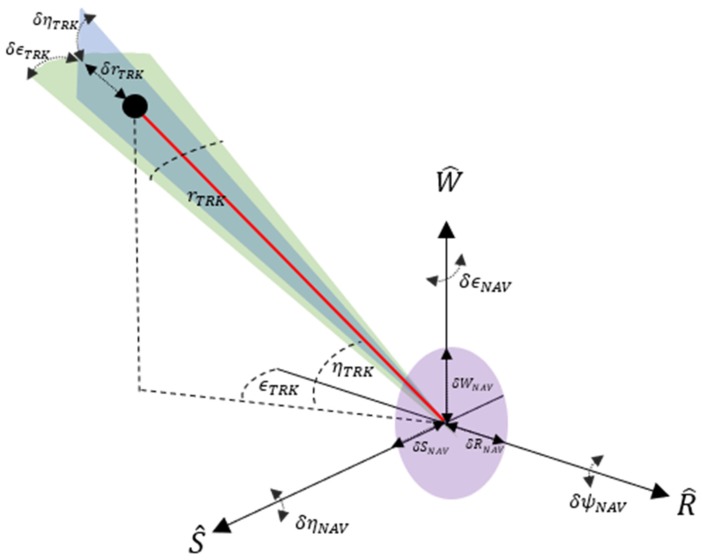
Space-based observation geometry and measurement deviations in RSW frame.

Based on proposed MMW SBR systems and documented GPS system performance, we outline here a mathematical framework to combine tracking and navigation uncertainty with the aim providing a rigorous methodology to describe and analyse the position uncertainty of a tracked RSO from a SBSS Radar platform. As with the ground-based scenario, we focus on the case of representing uncertainty expressed in a common satellite coordinate system to a convenient Earth-Centred Inertial (ECI) reference frame. In this case, the RSW coordinate system is used for both navigation and tracking observations, where the MMW radar (TRK) and GNSS (NAV) system is assumed to be centered about the origin. The reference geometry and the key symbols for this scenario are introduced in Figure 5 and Figure 6.

To quantify the total error about the RSO position, we reformulate the framework described in Section 2.2 as an Errors in-variable model following the Gauss–Helmert method [33,34]. In this case, we assume the attitude error of the spacecraft is zero ($\delta \epsilon_{NAV}=0$, $\delta \eta_{NAV}=0,\;\delta \psi_{NAV}=0)$. The generic Gauss–Helmert form consists in resolution of the following equation system:

(42) F(X,l)=0

where X, $\hat{l}$ are estimated parameters and observation vector respectively. The linearized form of the previous equation is:

(43) Aδ+Br+w=0

where $A=\frac{\partial F}{\partial \hat{X}}$ and $B=\frac{\partial F}{\partial l}$ are the matrix of partial derivates with respect to X, l and ***w*** is the misclosure vector. δ and r, the parameter and observation correction vector respectively, are:

(44) $$\begin{array}{c} \hat{\delta }=-{\left(A^{T}MA\right)}^{-1}A^{T}Mw\\ \hat{r}=-C_{r}B^{T}M\left(A\delta +w\right) \end{array}$$

where $M={\left(BC_{r}B^{T}\right)}^{-1}$ and $C_{r}$ is the covariance matrix of the observations. We obtain the covariance matrix of parameters:

(45) $$C_{NAV+TRK}={\left(A^{T}MA\right)}^{-1}$$

Equation (43) is then:

(46) $$F\left(X,l\right)=X_{D}-M_{RSW\to ECI}X_{TRK}-R_{ECI}=0$$

where:

$X_{D}=\left[\begin{array}{c} X_{D}^{ECI}\\ Y_{D}^{ECI}\\ Z_{D}^{ECI} \end{array}\right]$ and $R_{ECI}=\left[\begin{array}{c} X_{h}^{ECI}\\ Y_{h}^{ECI}\\ Z_{h}^{ECI} \end{array}\right]$ are the RSO and spacecraft position in ECI frame
$X_{TRK}=\left[\begin{array}{c} -r_{TRK}cos\left(\epsilon_{TRK}\right)cos\left(\eta_{TRK}\right)\\ r_{TRK}cos\left(\epsilon_{TRK}\right)sin\left(\eta_{TRK}\right)\\ r_{TRK}sin\left(\epsilon_{TRK}\right) \end{array}\right]$ is the nominal position of the target in RSW frame.
$l={\left[r_{TRK},\epsilon_{TRK},\eta_{TRK},x_{NAV},y_{NAV},z_{NAV}\right]}^{T}$ is vector of estimated observations, and
$C_{r}=\left[\begin{array}{cccccc} \sigma_{r_{TRK}}^{2} & 0 & 0 & 0 & 0 & 0\\ & \sigma_{\epsilon_{TRK}}^{2} & 0 & 0 & 0 & 0\\ & & \sigma_{\eta_{TRK}}^{2} & 0 & 0 & 0\\ & & & {\sigma_{x_{NAV}}}^{2} & \sigma_{{xy}_{NAV}} & \sigma_{{xz}_{NAV}}\\ & & & & {\sigma_{y_{NAV}}}^{2} & \sigma_{{yz}_{NAV}}\\ Sym & & & & & {\sigma_{z_{NAV}}}^{2} \end{array}]=[\begin{array}{cc} {Q_{TRK}}_{SPH}^{RDR} & 0\\ 0 & {Q_{NAV}}_{CART}^{ECI} \end{array}\right]$
  is the covariance matrix of observations.

The assumption is made that all navigation and tracking observations errors are independent, so covariance terms between the 2 observation sets in the matrix $C_{r}$ are set to zero. However, covariance terms between navigation uncertainty exist due to the transformation from the RSW to ECI coordinate frame described by Equation (18). With $A_{\left(3\times 3\right)}=𝟙$, the covariance matrix of observation is then computed by:

(47) $$C_{NAV+TRK}{}_{\left(3\times 3\right)}=\left(BC_{r}B^{T}\right)$$

## 5. Results

The aim of both scenarios was to demonstrate an effective framework for measurement uncertainty analysis while gaining a deeper understanding on the limits of a normally distributed representation of the uncertainty of the measurement models. In the first scenario, radar design parameters were selected based on ground-based radar tracking stations in the SSA SST network where in the second case radar parameters were selected from proposed spaceborne MMW Radar designs for larger orbiting platforms such as the ISS. As described in Section 4, the second case implements an error in variables model under a Gauss–Helmert formulation to combine both tracking the navigation measurements when determining the total position uncertainty of the tracked RSO. In doing so, navigation measurements are assumed to be provided by an on-board GNSS system where corresponding uncertainty values are taken from a LEO GPS accuracy experiment found in the literature [35]. Table 4 outlines the specific radar parameter and nominal tracking measurements (azimuth and elevation) values for both cases and the spacecraft orbital parameters (at measurement epoch) and associated uncertainty values within the RSW frame. To reflect the advantages of spaceborne MMW radar <10 cm RSO size was selected for the simulation, as opposed to the larger debris sizes (>10 cm) which have been chosen for the ground station scenario.

To test the covariance realism of the total position uncertainty the RSO the average Mahalanobis distance metric and Cramer–von Mises test statistic outlined in Section 2.3 is computed. Adapting the test procedure outlined in [10] for a sensor level analysis, the following steps are performed for both cases:

1. Define a range to target and debris size, calculate the performance of the radar system and fuse the tracking + navigation errors using the approach outline in Section 2.1
2. Generate N Monte Carlo points based on the measurement model performance as described in Section 2.3. (10,000 points were chosen in the case of these simulations)
3. Calculate the corresponding average Mahalanobis distance metric (AMD) and Cramer–von Mises (CVM) goodness of fit statistic.
4. Repeat steps 1–3 for every range to target for each RSO size.
5. Plot the averaged uncertainty metric (AMD) and the Cramer–von Mises test statistic versus range to target for each tracked RSO size
6. Determine the range to target when the averaged uncertainty metric and the Cramer–von Mises test statistic first pierce a pre-defined confidence interval (Table 2)– and declare that the covariance realism has broken down under the corresponding sensor performance.

**Figure 7:**
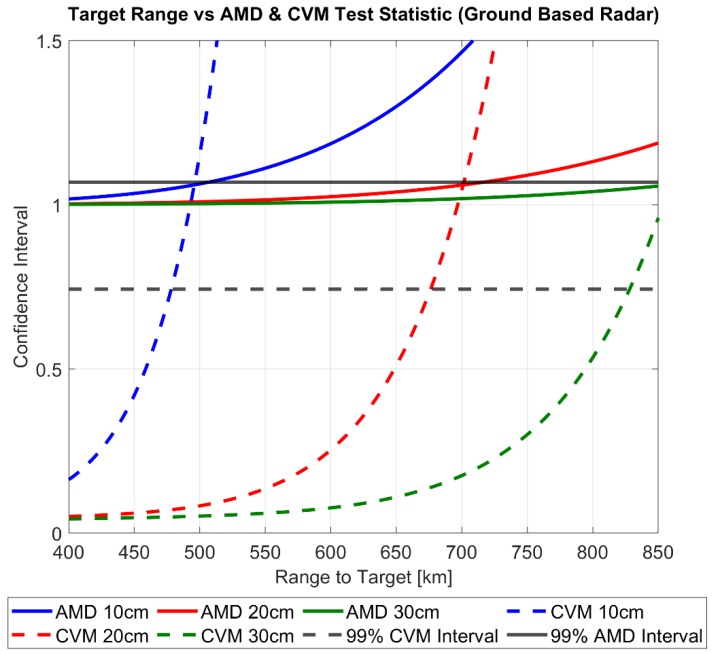
Covariance realism of theoretical uncertainty volume as a function of range to target for Ground-Based Radar.

**Figure 8:**
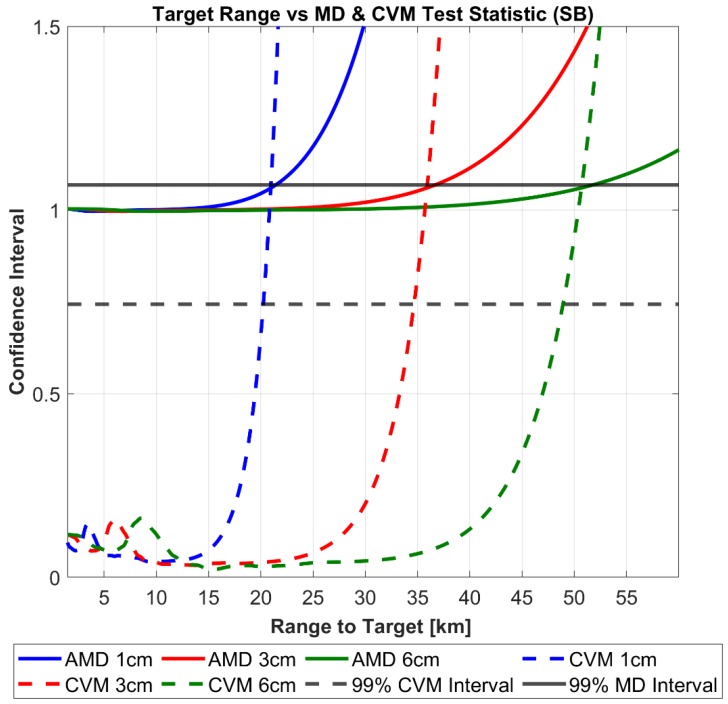
Covariance realism of theoretical uncertainty volume as a function of range to target for Space-Based Radar.

Figure 7 and Figure 8 displays the results of the above uncertainty realism test procedure which can be interpreted as follows: The calculated degree of the CVM test and AMD metric are plotted for each range to target as well as the confidence interval for each. Until the first point of intersect from either realism test and the corresponding confidence interval, the uncertainty distribution can be assumed to represent the calculated RSO covariance matrix under the chosen level of confidence. In both the ground and space-based cases, a confidence level of 99% was chosen arbitrarily. Both figures demonstrate that for all tracked RSO sizes, the CVM test statistic with the corresponding confidence interval provides a more restrictive statistical measure, when compared against the first-moment AMD metric. This is not a surprising result as the CVM test statistic is determined from the empirical CDF measurement model, giving more indication on the actual shape, size and orientation of the distribution. In turn the CVM test can distinguish finer discrepancies between the empirical (CDF) and the theoretical uncertainty distribution (covariance) when compared to the AMD metric. Table 5 outlines the difference in the range to target when between the CVM test statistic and AMD metric at the 99% confidence interval.

**Figure 9:**
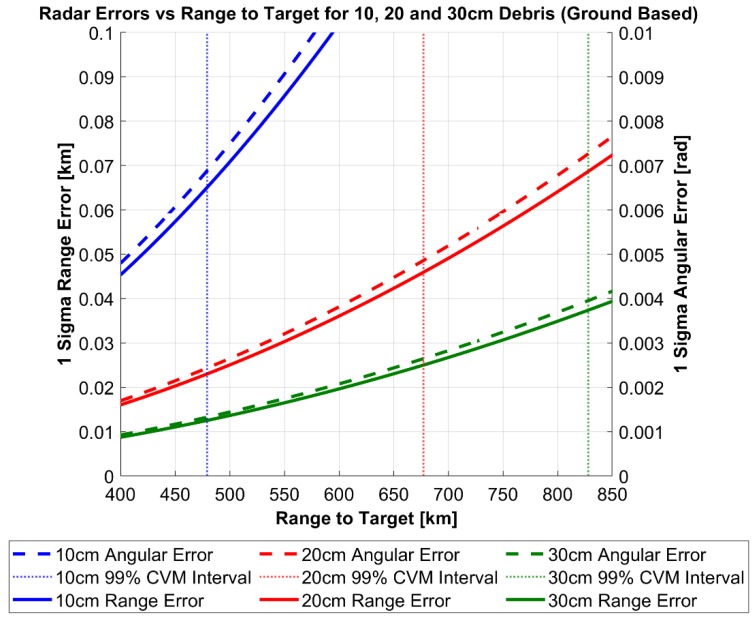
Space Based Radar range and angular errors as a function of range to target.

**Figure 10:**
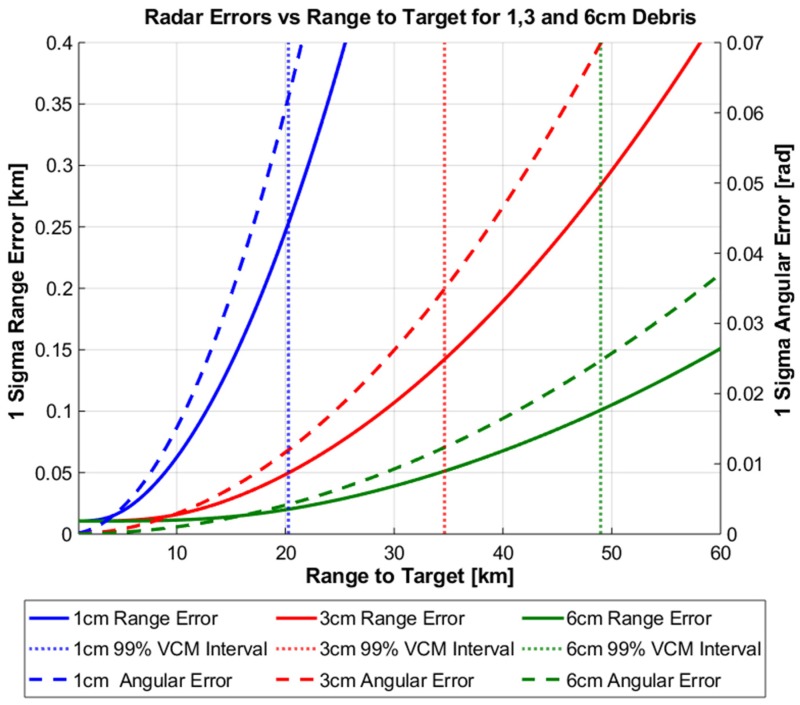
Ground Based Radar range and angular errors as a function of range to target.

Due to the significant impact on the calculated SNR of the radar system, assessing the covariance realism in relation to the specific range to target and debris size provide a practical relationship to defining an acceptable magnitude of measurement errors. Figure 9 and Figure 10 illustrate this relationship for the ground and space-based case, where the magnitude of range and angular errors and corresponding 99% CVM interval are plotted against the range-to-target for each debris size.

**Figure 11:**
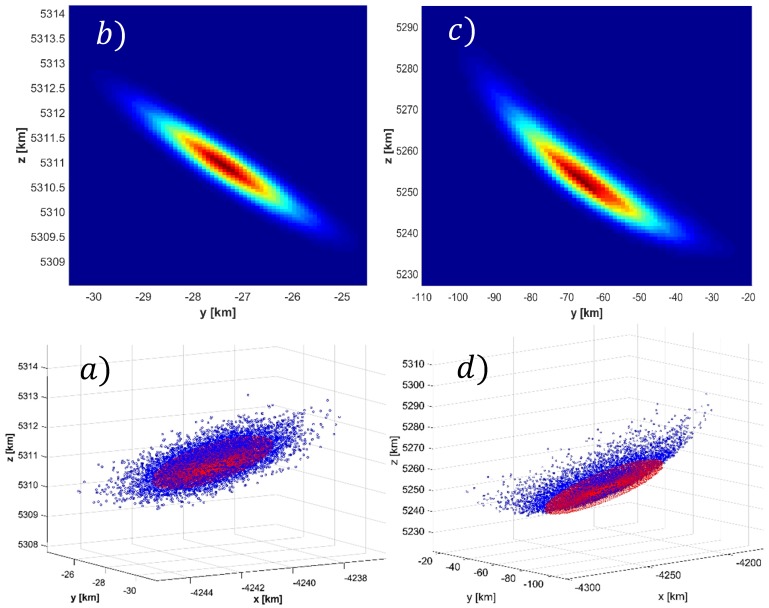
Monte Carlo generated distribution and corresponding contour maps for the 6 cm debris. Clockwise from bottom left: (**a**) 99%CVM Monte Carlo distribution (**b**) 99%CVM Contour Map, (**c**) >>99% CVM Confidence Interval Contour Map (**d**) >>99% CVM Confidence interval Monte Carlo distribution.

Mathematically, the confidence interval represents the point at which the original curvilinear distribution described by the radars spherical uncertainty can no longer be truthfully represented as rectilinear covariance within the cartesian ECI system. To illustrate this point further; Figure 11a shows the generation of Monte Carlo points used to generate the empirical distribution for the 6 cm RSO at the 99% CVM confidence interval for the 6 cm debris size. The calculated Cartesian covariance matrix inflated to 3 sigma is then overlaid as an ellipsoid centered about the nominal RSO position. As expected, the corresponding contour map (Figure 11b), illustrates that the Monte Carlo points conform to a rectilinear Gaussian distribution and therefore the corresponding uncertainty can be represented in terms of covariance within the ECI cartesian frame. Conversely, Figure 11c,d illustrate the distribution corresponding to range to 6 cm target of far-beyond the 99% CVM confidence interval. The distribution is now morphed from an ellipsoidal shape to a “bananoid”, a curvilinear gaussian distribution inherent to the radar measurement uncertainty model. Although this demonstrates the extreme case, meaning practically that the radar would not be used under these conditions due to the large uncertainty of the measurements, the figures aims to show physically what it means when the distribution becomes non-gaussian at the sensor level (in the rectilinear sense) and therefore cannot be described in terms of cartesian covariance. 

As previously highlighted, the importance of quantifying uncertainty at the sensor level is to meet the required assumptions of covariance realism for SSA activities such as orbit determination and uncertainty propagation for RSO collision probability analysis and subsequent avoidance activities. In effect, covariance realism at the sensor level provides means of covariance “fidelity” to these processes. Previously published studies on covariance realism for orbital propagation demonstrate that the initial AMD and CVM metric should tend unity and (1/12k) respectively to demonstrate that a large enough Monte Carlo sample size of the initial covariance matrix (of RSO position uncertainty) has been taken. Nonetheless, the sensor level analysis performed in this paper show that the initial covariance matrix used for these analyses may, in fact, vary in its actual realism/gaussianity if its intrinsic observation uncertainties have been mapped from its original coordinate system. For example, if an observation is taken under a certain tracking performance (in the case of this paper, RSO size and range-to-target), the subsequent covariance goodness of fit determined by the CVM test will lay somewhere along plot as shown in Figure 7 and Figure 8. Analysis of the effect of varying gaussianity as inputs to typical SSA analysis(orbit determination, probability of collision) is beyond the scope of this paper and will be addressed in future research.

Turning attention now specifically to the second case of an SBSS platform. Using the Gauss–Helmert errors in variables framework a measurement model was produced that combines both navigation and tracking errors to generate a position uncertainty of the tracked RSO. We are interested in identifying the influence of the navigation error on the total position uncertainty and any effect on the covariance realism tests described previously. In doing so, plotting the range to target against the ratio between NAV + TRK ($Q_{TOT}$) and TRK ($Q_{TRK}$) uncertainty provides an indication of the total effect of the navigation error on the total uncertainty of the RSO. This is done by taking the eigenvalues of each respective covariance matrix ($Q_{TOT},Q_{TRK})$ and summing them in an RSS manner, where the ratio between the two is then calculated.

(48) $${TOT}_{RSS}=\sqrt{\lambda_{1}{}_{Q_{TOT}}+\lambda_{2_{Q_{TOT}}}+\lambda_{3_{Q_{TOT}}}}$$

(49) $${TRK}_{RSS}=\sqrt{\lambda_{1}{}_{Q_{TRK}}+\lambda_{2_{Q_{TRK}}}+\lambda_{3_{Q_{TRK}}}}$$

**Figure 12:**
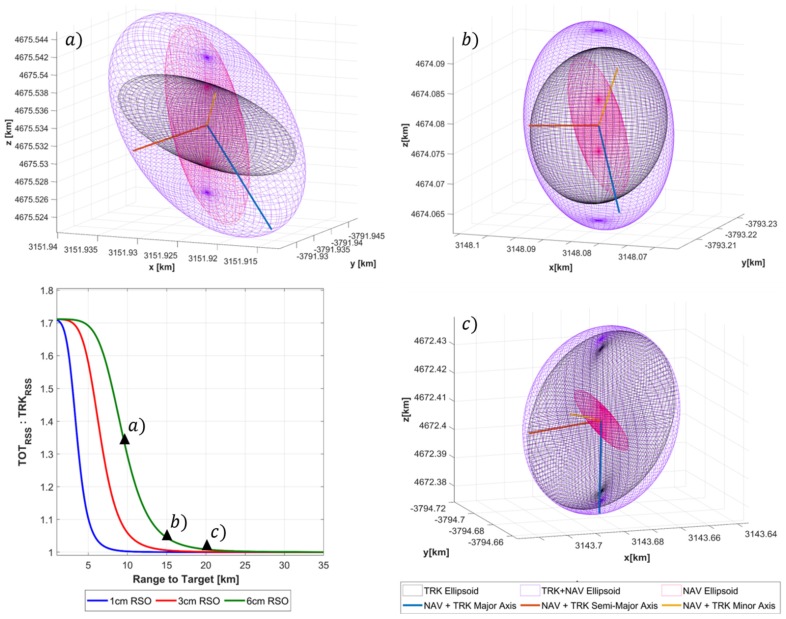
Effect of Navigation errors on total RSO uncertainty of tracked debris (clockwise from top right:). (**a**)10 km (**b**)15 km, (**c**)20 km range to target. In each figure the magenta, black and purple ellipsoids represent the Navigation, Tracking, and Total (NAV+TRK) uncertainty volumes respectively.

From Figure 12a, it is clear the navigation error uncertainty has a strong influence on the total error uncertainty volume at close target ranges, however as the range increases the ratio between the two uncertainty volumes decreases asymptotically to 1. This result is expected as the navigation error is assumed fixed during observation however the calculated radar performance is dynamic and heavily dependent on the range to target. Not surprisingly, Figure 7 demonstrates that navigation error has.

A significantly larger influence when a higher performance radar configuration is used, which in the case of the SNR dependent error corresponds to a larger size RSO being tracked. Figure 12b–d graphically illustrates the influence of navigation uncertainty error on the total uncertainty size and orientation at the 10, 15, 20 km range to a 6 cm target. At each range value, the navigation (NAV), tracking (TRK) and total (NAV + TRK) are represented as magenta, black and purple respectively. Regarding the effect of navigation uncertainty has the covariance realism, we can see that that the navigations uncertainty region of influence as defined by the ratio between ${TOT}_{RSS}$ and ${TRK}_{RSS}$ asymptotes to unity well before the range to target of the corresponding confidence interval. This indicates that under these specific simulation parameters the navigation error is not a limiting factor in maintaining gaussianity assumptions. However, on referring to Figure 8, it is shown that under close range where the navigation error is a dominant, oscillations of the CVM test occur for all debris sizes. Further research will address these findings and aim to address under what conditions could be detrimental to covariance realism.

The aleatoric benefits of the presented framework and covariance realism studies are quite clear – by accurately modelling all prominent sources of error that contribute to RSO uncertainty a realistic uncertainty regarding the position of an RSO is determined *(Parameter value uncertainty)*. Additionally, by addressing under what specific sensor performance RSO uncertainty (gaussian) assumptions maintain realism, *Model-Based Uncertainty* is also explicitly quantified providing both a top down and bottom up methodology to sensor performance requirements. Nonetheless, future Trusted Autonomous Systems (TAS): will be required to address not only the aleatoric elements discussed but also epistemic uncertainty. Importantly, within the context of future STM/SSA and the CHMI2 concept, uncertainty quantification translates into the confidence-building measures required to inform a trusted closed loop decision-making process between the space analyst and the machine (system autonomy).

## 6. Conclusions and Future Research

Ground and space-based surveillance platforms will form a necessary aspect in the categorisation and maintenance of resident space object databases, required for timely and effective SSA operation within a future Space Traffic Management (STM) system. Nonetheless, for this to be a viable and useful asset, it is necessary that a sensor focused approach is taken when determining position uncertainty of Resident Space Objects (RSO). Through a representative case study of a Ground-Based and Spaceborne MMW radar, this paper aimed to demonstrate an error model that captures both the navigation and tracking errors in determining RSO position uncertainty. Practically, the results show that sensor performance must be determined under different tracking conditions to uphold uncertainty realism assumptions and support key SSA decision-making processes. Nonetheless, an effective STM system will require a deeper understanding of the orbital environment, where forms of traditional uncertainty in space object position and trajectory must extend to space object operational intent, modes, and other important decision-making criteria. In effect, this requires that decision making tools must evolve from addressing not only parameter (inputs) and model-based uncertainty (models that process inputs) but also the epistemic uncertainty within the on-orbit environment Future research will focus on addressing the human centric cyber-physical system challenges associated with a future STM system, while exploring promising Low SWAP-C space surveillance sensors and cooperative net-centric data-sharing systems for SSA.

## Figures and Tables

**Table 1:** Overview of Space Surveillance and Tracking (SST) ground-based radar systems {Choi, 2017 #55}.

Ground-Based Radar System	Devices	Description	Location
AN/FPS-85	UHF Phased-array radar	Maximum peak power: 30 MW, it can detect 1.0 $m^{2}$ objects in geosynchronous orbits	Florida, USA
Globus II	X-Band mono-pulse radar with 27 m parabolic dish antenna	Track spacecraft of all type up to range of 4000 km	Vardo, Norway
TIRA	L-band and Ku-band radar using 34 m parabolic reflector	Radar images of space objects at a distance up to 20,000 km	Watchberg, Germany

**Table 2:** The categorisations of collective uncertainty for autonomous systems.

Category	Type of Uncertainty	Description
**Aleatoric uncertainty**	Parameter Value Uncertainty	Uncertainty in scenario Inputs (Independent variables)
	Model-Based Uncertainty	Uncertainty in the underlying models that process inputs
**Epistemic uncertainty**	Uncertainty of Focus	Uncertainty due to a known lack of knowledge about the environment
	Complexity of Uncertain Factors	When the environment is sufficiently complex a machine cannot overview the set off all possible true states

**Table 3:** Confidence interval for Cramer–von Mises (CVM) [12] and Mahalanobis Distance (MD) for ∞ samples.

	90%	95%	99%	99.9%
AMD	[0.9655,1.0457]	[0.9578,1.0534]	[0.9427,1.0685]	[0.9106,1.1006]
CVM	[0,0.3430]	[0,0.46136]	[0,0.74346]	[0,1.16204]

**Table 4:** Ground and Space-based tracking scenario inputs.

**Spacecraft Position**	**a = 6829 km**	**e = 0.00001**	**i = 51.6°**	**ω = 90°**	**Ω = 90°**
**Navigation Error**					
Radial (R) $\sigma_{R_{NAV}}$	13.81 m				
In-Track (S) $\sigma_{S_{NAV}}$	4.15 m				
Cross-Track (W) $\sigma_{W_{NAV}}$	3.0 m				
**Nominal Tracking Angle**	**Space-Based Radar**			**Ground-Based Radar**	
$\epsilon_{TRK}$	45°			45°	
$\eta_{TRK}$	45°			45°	
**Fixed Radar Parameters**					
Frequency	95 GHz (W band)			442 MHz (UHF)	
Peak transmit power	1200 W			36 MW	
Beamwidth	0.2°			1.3°	
Aperture Dimension	1.0 m			58.0 m	
Noise Figure	4.5 dB			4.5 dB	
Radar pulse duration	1 μs			1 μs	
Transmit antenna Gain	58 dBi			48 dBi	
**Varied Parameters**					
Debris Diameter	1, 3, 6 cm			10, 20, 30 cm	
Range to Target $r_{TRK}$	1:60 km			1:850 km	

**Table 5:** Max Range-to-Target for 99% Average Mahalanobis Distance (AMD) and CVM Covariance Realism Test Statistic.

	Space-Based Tracking			Ground-Based Tracking		
RSO Size	1 cm	3 cm	6 cm	10 cm	20 cm	30 cm
AMD range [km]	21.393	36.689	51.825	505.54	716.61	876.80
CVM range [km]	20.027	34.618	48.958	479.15	677.03	827.80
Δ range to target [km]	1.115	2.071	2.867	26.38	39.57	49
